# Inferring pleiotropy by network analysis: linked diseases in the human PPI network

**DOI:** 10.1186/1752-0509-5-179

**Published:** 2011-10-31

**Authors:** Thanh-Phuong Nguyen, Wei-chung Liu, Ferenc Jordán

**Affiliations:** 1 The Microsoft Research - University of Trento, Centre for Computational and Systems Biology, Povo/Trento, Italy; 2 Institute of Statistical Science, Academia Sinica, Taipei, Taiwan

## Abstract

**Background:**

Earlier, we identified proteins connecting different disease proteins in the human protein-protein interaction network and quantified their mediator role. An analysis of the networks of these mediators shows that proteins connecting heart disease and diabetes largely overlap with the ones connecting heart disease and obesity.

**Results:**

We quantified their overlap, and based on the identified topological patterns, we inferred the structural disease-relatedness of several proteins. Literature data provide a functional look of them, well supporting our findings. For example, the inferred structurally important role of the PDZ domain-containing protein GIPC1 in diabetes is supported despite the lack of this information in the Online Mendelian Inheritance in Man database. Several key mediator proteins identified here clearly has pleiotropic effects, supported by ample evidence for their general but always of only secondary importance.

**Conclusions:**

We suggest that studying central nodes in mediator networks may contribute to better understanding and quantifying pleiotropy. Network analysis provides potentially useful tools here, as well as helps in improving databases.

## Background

The systems perspective on complex biological systems emphasizes that individual genes act in genetic networks and individual proteins play their roles in protein-protein interaction (PPI) networks [1]. There is increasing interest in these networks, as their analysis helps to understand the relationship between the components (i.e. genes, proteins) and how these are positioned in the whole system. Well-connected hubs seem to be of high functional importance [2,3]. Consequently, studies on diseases based on PPI networks had the starting point by analysing the centrality of disease proteins. Genes associated with a particular phenotype or function are not randomly positioned in the PPI network, but tend to exhibit high connectivity; they may cluster together and can occur in central network locations [4,5].

Beyond focusing on the number of neighbours of graph nodes (their degree), wider neighbourhoods, indirect effects and larger subsets of nodes can also be analyzed by the rich arsenal of network analytical tools. This non-local information may help, for example, to quantify the structural relationships between different sets of proteins. In an earlier paper [6], we have determined proteins that mediate indirect effects between sets of proteins causing five diseases in the human PPI network. Their mediator role was quantified and they were ranked according to structural importance. Their functional role may be of high interest, as proteins involved in certain pairs of diseases have no direct interactions among them [6]. These findings motivated an appealing problem: „which proteins connect diseases in the human PPI network?".

To be connected to diverse regions of the PPI network may lend a functionally pleiotropic character to a protein in a classical, genetic sense: it has been demonstrated that high connectivity correlates well with pleiotropic effects [7,8]. The most central mediators are especially important in connecting apparently distant nodes in the human PPI network. Specific network positions may render strange but characteristic behaviour (expression pattern) to different proteins [9,10]. Instead of being exceptional, these epistatic effects may be of primary importance in physiology [11] and in better understanding animal development and adaptation.

In this paper, (1) we compare two interaction networks of mediators (mediating indirect effects between heart disease and obesity, and between heart disease and diabetes), (2) we analyse the structure of these two networks and their aggregated total network, (3) we study the overlap between the two mediator networks, and (4) we infer biological functions for some proteins and provide supporting literature data. All in all, we illustrate that network analysis is an excellent tool for identifying pleiotropy and epistasis from complex networks extracted from multiple databases.

## Results

### Network analysis

**Figure 1:**
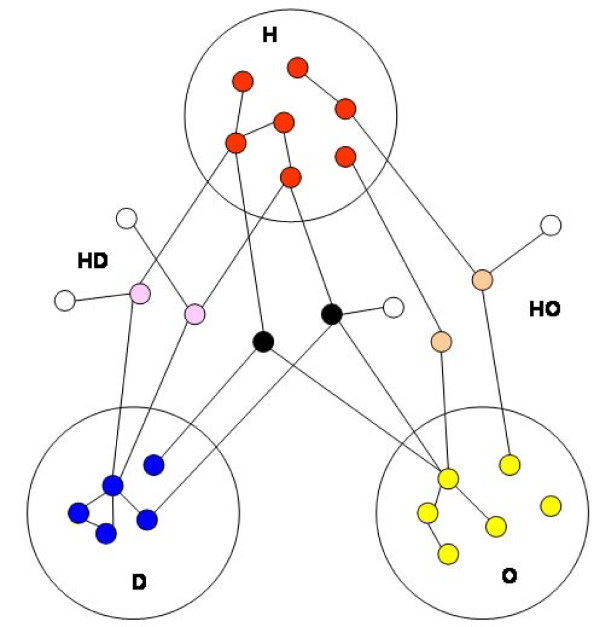
**The HD and HO mediator networks and their subnetworks**. Red, blue and yellow proteins are involved in three diseases (H: heart diseases, D: diabetes, O: obesity). Pink proteins mediate indirect effects between the red and the blue ones, while orange proteins mediate between the red and the yellow ones. Black proteins mediate between both pairs. White proteins are the non-mediator neighbours of the mediator proteins. We analyzed five networks: the HD mediator network (pink and black nodes with their white neighbours), the HO mediator network (orange and black nodes with their white neighbours), the total mediator network (pink, orange and black nodes with their white neighbours), the subnetwork of interactions among HD mediators (pink and black nodes) and the subnetwork of interactions among HO mediators (orange and black nodes).

**Figure 2:**
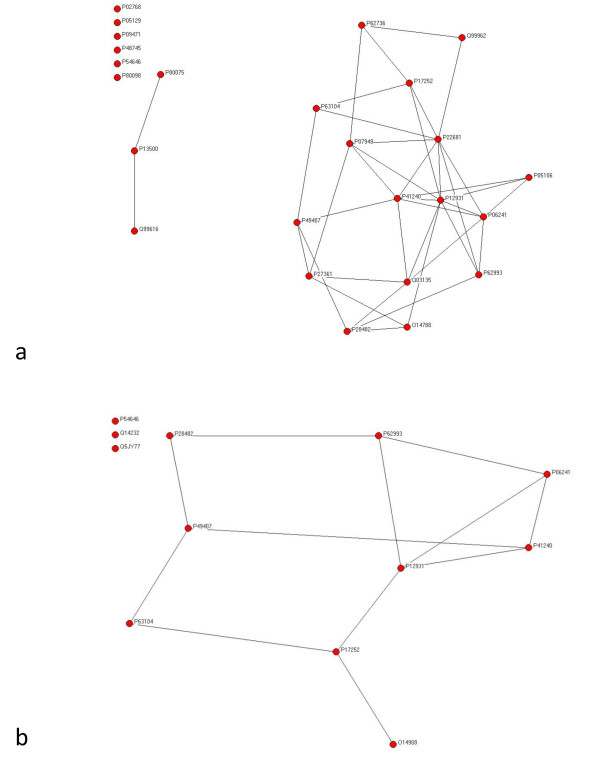
**Subgraphs of the HD (a) and HO (b) networks, showing the interactions only between HD and HO mediators, respectively**.

**Figure 3:**
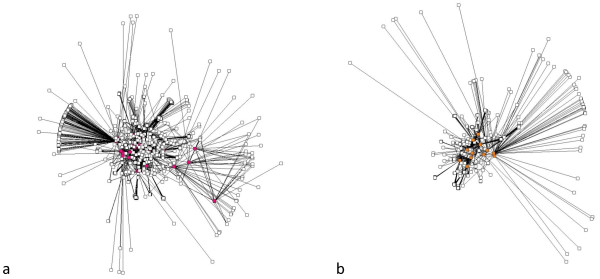
**The HD (a) and HO (b) networks: pink and orange nodes are the HD and HO mediators, respectively, while the white nodes are their non-mediator neighbours**.

**Figure 4:**
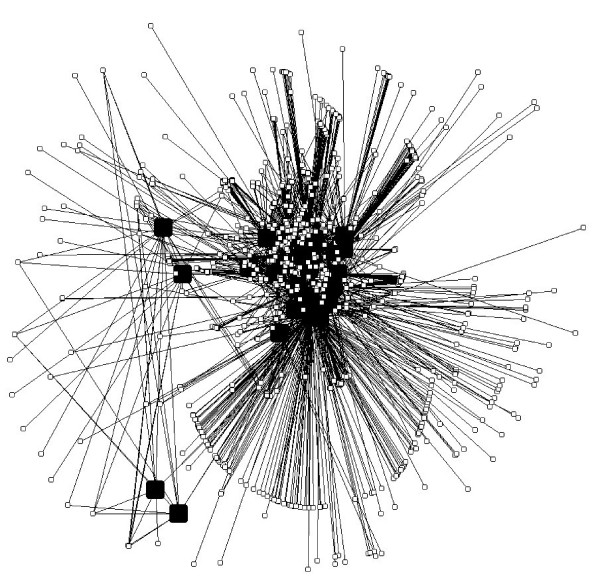
**The total network: black nodes are the HD or HO mediators, while the white nodes are their non-mediator neighbours**.

We obtained 9 proteins involved in heart diseases (H), as well as 44 and 20 involved in diabetes (D) and obesity (O), respectively. The HD network contains N = 2142 nodes and L = 3537 links, while the HO network contains N = 1746 nodes and L = 2567 links and the total network contains N = 2221 nodes and L = 3686 links. Figure 1 provides a schematic illustration for how the networks had been constructed (see Methods). Figure 2 shows the relationships between mediator proteins in the HD (Figure 2a) and the HO (Figure 2b) networks. The HD network (Figure 3a) contains 25 HD mediators and their 2117 neighbours and the HO network (Figure 3b) contains 12 HO mediators and their 1734 neighbours. In the „total" network (Figure 4), 9 shared mediators appear, so it contains only 28 mediator proteins. In this total network, 1667 nodes are present in both the HD and the HO network, 475 only in the HD and 79 only in the HO network.

**Figure 5:**
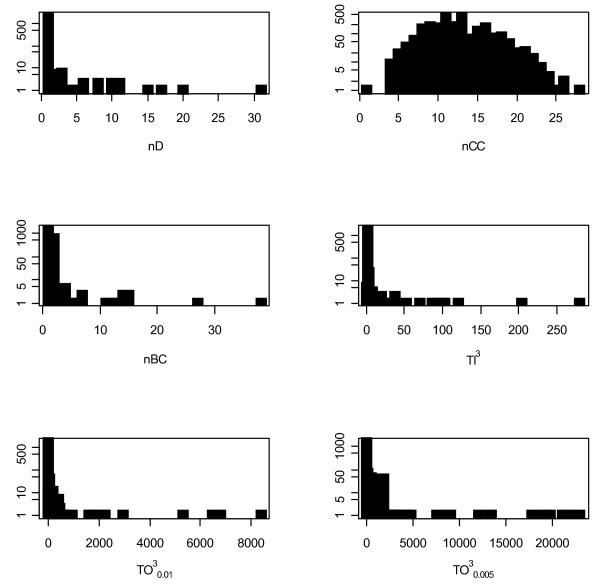
**The distributions of nodal index values in the total network**.

The distributions of individual structural indices are very similar for all of the three analyzed networks. Additional file 1 shows all values of the six network indices for all nodes in the three networks. Figure 5 shows these distributions only for the total network. We can observe that almost all indices follow a strongly left-skewed distribution where only a few nodes are extremely important. While degree (*D)*, topological importance (*TI*) and betweenness centrality (*BC*) have really only one or a few hubs, topological overlap (*TO*) indicates several key nodes. Closeness centrality (*CC*) has a unimodal, normal-like distribution.

For each network, there seem to be strong and positive rank correlation between all centrality indices but not for the overlap indices (*TO*^3^_0.01 _and *TO*^3^_0.005_). *TO *indices correlate positively and weakly with other centrality indices whereas they correlate negatively and weakly with *CC *(see Table 1). *D *best correlates with *TI^3^*. The *TO *measure offers different, complementary information than the centrality indices.

**Table 1:** Correlations between indices of the real networks.

HD	nCC	nBC	TI^3^	TO^3^_0.01_	TO^3^_0.005_
nD	0.713	0.816	0.862	0.216	0.309
nCC		0.59	0.516	-0.092	-0.051
nBC			0.73	0.249	0.324
TI^3^				0.265	0.265
TI^3^_0.01_					0.717
**HO**	nCC	nBC	TI^3^	TO^3^_0.01_	TO^3^_0.005_
nD	0.737	0.72	0.82	0.069	0.187
nCC		0.579	0.377	-0.225	-0.138
nBC			0.625	0.181	0.249
TI^3^				0.262	0.302
TI^3^_0.01_					0.836
total	nCC	nBC	TI^3^	TO^3^_0.01_	TO^3^_0.005_
nD	0.704	0.819	0.862	0.169	0.27
nCC		0.585	0.47	-0.186	-0.132
nBC			0.732	0.223	0.307
TI^3^				0.272	0.29
TI^3^_0.01_					0.775

The Spearman rank correlation coefficients between each pair of centrality indices for the HD and HO networks as well as the total network.

Table 2 summarizes the results of the randomization test (note that only the means are shown in the table, for simplicity). The observed rank correlation coefficients are all significantly lower than those for the random networks (with 95% confidence interval). This suggests that there are stronger rank correlations between different centrality indices in the random networks, in comparison to the results obtained from the HD, HO and total networks. One possible explanation for this discrepancy is that, beyond the mathematical properties, real networks are structured also by biological constraints. Thus, different centrality indices can capture different aspects of network topology, therefore correlation between different indices are weaker for real networks. This provides more support on using various network indices to capture different topological properties embedded in real networks.

**Table 2:** Correlations between indices of the randomized networks.

HD	nCC	nBC	TI^3^	TO^3^_0.01_	TO^3^_0.005_
nD	0.82	0.96	0.935	0.929	0.93
nCC		0.878	0.621	0.836	0.887
nBC			0.87	0.918	0.927
TI^3^				0.79	0.773
TI^3^_0.01_					0.942
**HO**	nCC	nBC	TI^3^	TO^3^_0.01_	TO^3^_0.005_
nD	0.796	0.954	0.926	0.919	0.91
nCC		0.853	0.571	0.857	0.894
nBC			0.849	0.913	0.908
TI^3^				0.749	0.723
TI^3^_0.01_					0.95
**total**	nCC	nBC	TI^3^	TO^3^_0.01_	TO^3^_0.005_
nD	0.839	0.962	0.936	0.931	0.933
nCC		0.893	0.647	0.864	0.901
nBC			0.874	0.921	0.931
TI^3^				0.792	0.779
TI^3^_0.01_					0.945

The mean Spearman rank correlation coefficients between each pair of centrality indices obtained from 1000 random networks of the same size as the HD, HD and the total network.

### Biological results

We now examine more closely the rank order of the top nodes in each network. The degree ranks for the three networks are almost identical (see Tables 3, 4 and 5). The most central nodes are P62993 (Growth factor receptor-bound protein 2), P63104 (14-3-3 protein zeta/delta) and P06241 (Tyrosine-protein kinase Fyn). The 9 shared proteins rank in the same order in HD and HO and there is no change in rank order also in the total network. In the HO network, the 12 mediators lead the ranking, and then come their neighbours. However, in the HD rank (and also in the total network), there is one non-mediator protein in the top 26 of the rank (among the 25 HD mediators); this is P00533 (Epidermal growth factor receptor) in the 23^rd ^position.

**Table 3:** Centrality ranks for the HD network.

	nD		nCC		nBC		TI^3^		TO^3^_0.01_		TO^3^_0.005_
**P62993**	31.11	**P62993**	46.54	**P62993**	37.39	**P62993**	277.53	**P62993**	8066	**P12931**	20642
**P63104**	19.80	**P12931**	44.47	**P63104**	26.06	**P63104**	204.08	**P63104**	7107	**P62993**	19760
**P06241**	16.21	**P22681**	44.31	**P12931**	13.57	**P06241**	117.74	**P12931**	6893	**P06241**	19595
**P12931**	14.67	**P00533**	43.76	**P17252**	13.52	**P12931**	100.73	**P06241**	5541	**P63104**	18434
**P17252**	10.32	**P06241**	42.78	**P49407**	13.33	**P49407**	100.05	**P17252**	4025	**P49407**	16818
**P49407**	10.32	**Q13813**	42.67	**P06241**	12.97	**P62736**	95.47	**P28482**	2338	**P62736**	16177
**P62736**	9.81	**P00519**	42.01	**P62736**	11.69	**P17252**	90.13	**P07948**	2104	**P17252**	15588
**P28482**	8.97	**P21333**	41.75	**P02768**	10.94	**P02768**	86.69	**P62736**	1875	**P28482**	13598
**P27361**	7.43	**P63104**	41.74	**P28482**	9.30	**P28482**	66.61	**P27361**	1800	**P22681**	13337
**P02768**	7.19	**P17252**	41.69	**P22681**	5.71	**P27361**	52.43	**P49407**	1661	**P07948**	12088
**P22681**	5.42	**P07355**	41.48	**P27361**	5.35	**Q03135**	36.93	**P22681**	1634	**P27361**	9436
**P07948**	5.37	**P61978**	41.45	**Q03135**	4.67	**P22681**	36.86	**P51681**	1280	**Q03135**	9271
**Q03135**	4.58	**P28482**	41.39	**P07948**	3.38	**P07948**	35.10	**P05129**	955	**P41240**	7227
**P41240**	2.80	**Q02156**	40.82	**P09471**	2.25	**Q99962**	18.32	**P09471**	925	**P05129**	3574
**P05129**	2.43	**P29353**	40.77	**Q99962**	2.08	**P05106**	18.32	**O15303**	875	**P05106**	3532
**P05106**	2.38	**P07900**	40.70	**P05106**	2.02	**P09471**	18.02	**O15492**	875	**P98082**	1867
**Q99962**	2.01	**P62988**	40.68	**P41240**	1.44	**P05129**	17.42	**O15539**	875	**P51681**	1828
**P09471**	1.54	**O14939**	40.64	**P00533**	0.97	**P41240**	15.68	**O15552**	875	**Q99962**	1730
**P13500**	1.03	**P43405**	40.52	**Q9UBS5**	0.89	**P48745**	6.64	**O43566**	875	**P18031**	1574
**O14788**	0.79	**Q06124**	40.51	**P18545**	0.84	**P13500**	6.11	**O43665**	875	**P00533**	1521
**P80098**	0.79	**Q07889**	40.51	**P13500**	0.84	**P80098**	5.16	**O76081**	875	**O15492**	1504
**P48745**	0.61	**P06396**	40.29	**P05129**	0.82	**P54646**	5.08	**P04899**	875	**P04004**	1471
**P00533**	0.51	**P56945**	40.28	**P06396**	0.80	**O14788**	4.81	**P08913**	875	**P49757**	1471
**P80075**	0.51	**P06213**	40.19	**P48745**	0.76	**P80075**	2.53	**P16473**	875	**P16284**	1440
**P54646**	0.47	**O43707**	40.16	**P80098**	0.69	**Q99616**	2.42	**P18545**	875	**P29353**	1440
**Q99616**	0.47	**Q05655**	40.06	**P61981**	0.69	**P00533**	2.14	**P18825**	875	**Q05397**	1440
**P29353**	0.42	**P49407**	40.01	**P11532**	0.67	**P29353**	1.58	**P30542**	875	**Q99704**	1440
**P06213**	0.37	**Q15746**	40.00	**O14788**	0.64	**P17302**	1.51	**P32302**	875	**P31751**	1308
**P17302**	0.37	**P23528**	39.93	**Q92616**	0.62	**P43405**	1.44	**P34998**	875	**P02751**	1297
**P43405**	0.37	**Q00839**	39.93	**P02751**	0.59	**P06213**	1.43	**P35372**	875	**P06756**	1297

The rank of the most central 30 nodes in the HD network, based on the six importance indices analyzed.

**Table 4:** Centrality ranks in the HO network.

	nD		nCC		nBC		TI^3^		TO^3^_0.01_		TO^3^_0.005_
**P62993**	38.17	**P62993**	50.40	**P62993**	47.09	**P62993**	307.09	**P62993**	6637	**P62993**	14457
**P63104**	24.30	**P12931**	46.24	**P63104**	32.23	**P63104**	215.63	**P63104**	6333	**P63104**	14457
**P06241**	19.89	**P21333**	45.68	**P17252**	17.01	**P06241**	134.44	**P12931**	5744	**P49407**	14244
**P12931**	17.99	**P07355**	45.61	**P12931**	16.91	**P12931**	117.55	**P06241**	5056	**P17252**	12792
**P17252**	12.67	**Q13813**	45.61	**P49407**	16.71	**P49407**	108.09	**P17252**	3089	**P12931**	12396
**P49407**	12.67	**P22681**	45.42	**P06241**	16.12	**P17252**	106.48	**P28482**	2667	**P06241**	10742
**P28482**	11.00	**P00533**	45.40	**P28482**	13.75	**P28482**	88.40	**P49407**	1797	**P28482**	10633
**P41240**	3.44	**P61978**	44.96	**Q5JY77**	4.04	**Q5JY77**	26.59	**P41240**	1035	**P41240**	4498
**Q5JY77**	2.69	**Q07889**	44.56	**O14908**	3.32	**O14908**	22.70	**P07550**	244	**O14908**	3414
**O14908**	2.58	**Q13322**	44.56	**P41240**	2.03	**P41240**	19.48	**Q14232**	242	**Q5JY77**	3363
**Q14232**	0.97	**P06241**	44.20	**P07550**	1.51	**Q14232**	8.44	**P08913**	215	**P04629**	3281
**P54646**	0.57	**P11142**	44.16	**Q14232**	0.94	**P54646**	5.41	**P14866**	215	**P08588**	3281
**P00533**	0.34	**P07900**	44.01	**P08588**	0.73	**P07550**	1.40	**P18089**	215	**P98164**	3281
**P07550**	0.34	**P62988**	44.00	**P54646**	0.69	**P07900**	1.37	**P18825**	215	**P07550**	2835
**P07900**	0.34	**P63104**	43.46	**P21333**	0.59	**P00533**	1.32	**P81605**	215	**P08069**	1976
**P22681**	0.34	**P29353**	43.38	**P07900**	0.57	**P61978**	1.28	**O14908**	208	**O00222**	1872
**P29353**	0.34	**P35568**	43.32	**P07355**	0.51	**P22681**	1.28	**Q92793**	189	**O15534**	1872
**P61978**	0.34	**P00519**	43.29	**Q13813**	0.51	**P29353**	1.25	**P23508**	186	**O43193**	1872
**O14939**	0.29	**P56945**	43.29	**P61978**	0.51	**P21333**	1.19	**P05198**	185	**O43504**	1872
**P04049**	0.29	**P28482**	42.94	**Q9UQ35**	0.48	**P07355**	1.18	**P13667**	180	**O60518**	1872
**P06213**	0.29	**O43707**	42.74	**P00533**	0.47	**Q13813**	1.18	**P20042**	180	**O60925**	1872
**P07355**	0.29	**P11274**	42.74	**O43707**	0.46	**P04049**	1.12	**P49703**	180	**O75665**	1872
**P21333**	0.29	**P02545**	42.47	**P11274**	0.46	**Q02156**	1.12	**P49770**	180	**O95295**	1872
**P49023**	0.29	**P05783**	42.47	**P22681**	0.46	**Q05513**	1.12	**P52565**	180	**P08173**	1872
**P98082**	0.29	**Q16658**	42.47	**P04629**	0.46	**Q07889**	1.11	**Q13144**	180	**P08912**	1872
**Q02156**	0.29	**P17252**	42.45	**P23458**	0.41	**Q13322**	1.11	**Q9BYD3**	180	**P11229**	1872
**Q05513**	0.29	**P23458**	42.45	**P11142**	0.39	**P06213**	1.06	**Q9NR50**	180	**P20309**	1872
**Q07889**	0.29	**P04049**	42.41	**Q7KZI7**	0.39	**P08588**	1.05	**Q5JY77**	168	**P21452**	1872
**Q13322**	0.29	**Q02156**	42.41	**P30556**	0.39	**P49023**	1.03	**O00418**	165	**P25025**	1872
**Q13813**	0.29	**Q05513**	42.41	**Q07889**	0.37	**O14939**	1.02	**O00763**	165	**P25103**	1872

The rank of the most central 30 nodes in the HO network, based on the six importance indices analyzed.

**Table 5:** Centrality ranks in the total network.

	nD		nCC		nBC		TI^3^		TO^3^_0.01_		TO^3^_0.005_
**P62993**	30.23	**P62993**	46.11	**P62993**	36.84	**P62993**	278.83	**P62993**	8381	**P62993**	23017
**P63104**	19.10	**P12931**	44.07	**P63104**	25.10	**P63104**	201.37	**P12931**	6791	**P63104**	22032
**P06241**	15.81	**P22681**	43.81	**P12931**	13.34	**P06241**	118.70	**P63104**	6489	**P12931**	21112
**P12931**	14.28	**P00533**	43.04	**P17252**	13.07	**P12931**	101.69	**P06241**	5322	**P49407**	19777
**P49407**	10.00	**P06241**	42.16	**P49407**	12.99	**P49407**	98.76	**P17252**	2966	**P06241**	19449
**P17252**	9.96	**Q13813**	42.00	**P06241**	12.50	**P62736**	95.29	**P28482**	2240	**P17252**	19070
**P62736**	9.51	**P17252**	41.64	**P62736**	11.36	**P17252**	88.26	**P07948**	2084	**P62736**	17788
**P28482**	8.78	**P63104**	41.38	**P02768**	10.53	**P02768**	86.12	**P62736**	1818	**P22681**	13383
**P27361**	7.03	**P00519**	41.20	**P28482**	9.33	**P28482**	68.56	**P49407**	1784	**P28482**	13267
**P02768**	6.94	**P21333**	41.12	**P22681**	5.49	**P27361**	51.72	**P27361**	1734	**P07948**	12041
**P22681**	5.27	**P28482**	41.10	**P27361**	5.11	**P22681**	36.87	**P22681**	1628	**P27361**	8970
**P07948**	5.18	**P07355**	40.88	**Q03135**	4.51	**Q03135**	36.27	**P05129**	944	**Q03135**	8488
**Q03135**	4.37	**P61978**	40.67	**P07948**	3.22	**P07948**	35.12	**P41240**	677	**P41240**	7515
**P41240**	2.70	**Q02156**	40.28	**Q5JY77**	2.91	**Q5JY77**	24.85	**P13500**	559	**P09471**	4708
**P05129**	2.34	**P29353**	40.26	**O14908**	2.60	**O14908**	22.16	**Q03135**	552	**O14908**	4366
**P05106**	2.30	**O14939**	40.12	**P09471**	2.15	**Q99962**	18.30	**P08254**	525	**Q5JY77**	4319
**Q5JY77**	2.16	**P62988**	40.12	**Q99962**	2.01	**P05106**	18.15	**P03956**	511	**P08588**	4194
**O14908**	2.12	**P43405**	40.00	**P05106**	1.91	**P09471**	18.11	**P05106**	497	**P05129**	3488
**Q99962**	1.94	**Q06124**	39.99	**P41240**	1.45	**P05129**	17.16	**P80075**	444	**P05106**	3468
**P09471**	1.58	**O43707**	39.91	**P00533**	0.90	**P41240**	15.63	**P80098**	444	**P04629**	3288
**P13500**	0.90	**P07900**	39.86	**P07550**	0.89	**Q14232**	8.00	**Q99616**	438	**P98164**	3193
**O14788**	0.86	**Q07889**	39.82	**Q9UBS5**	0.83	**P48745**	6.63	**P51681**	435	**P41143**	2733
**P80098**	0.77	**P49407**	39.78	**P05129**	0.80	**O14788**	5.75	**O14788**	412	**P35372**	2721
**Q14232**	0.77	**P06213**	39.71	**P18545**	0.76	**P13500**	5.57	**O00590**	400	**P07550**	2551
**P48745**	0.59	**P56945**	39.58	**P06396**	0.75	**P80098**	5.25	**P39900**	400	**P49795**	2464
**P00533**	0.50	**P06396**	39.57	**Q14232**	0.74	**P54646**	5.07	**P41597**	400	**P08648**	2456
**P80075**	0.50	**Q15746**	39.50	**P48745**	0.73	**P80075**	2.48	**P51677**	400	**Q08116**	2452
**P54646**	0.45	**Q05655**	39.39	**O14788**	0.73	**Q99616**	2.38	**Q9NPB9**	400	**P41594**	2155
**Q99616**	0.45	**P11142**	39.31	**P13500**	0.71	**P00533**	2.12	**P32246**	373	**P08069**	1854
**P29353**	0.41	**P35568**	39.24	**P61981**	0.66	**P29353**	1.57	**Q16570**	373	**P98082**	1810

The rank of the most central 30 nodes in the total network, based on the six importance indices analyzed.

The betweenness ranks correspond quite well to the degree ranks with some exceptions. For example in the HD network, P06241 (Tyrosine-protein kinase Fyn) is three positions lower in betweenness ranking when compared to its degree rank position. In the HD network, instead of one, now five non-mediators are mixed with HD mediators in the top of the list, while some HD mediators such as Q99616 (C-C motif chemokine 13) lose their high degree-based rank completely. In contrast, the degree rank order seems to be consistent with its betweennes counterpart for HO and total networks.

Despite the large overlap between the HD and HO networks, the rank positions of HD and HO mediator proteins are quite different in the two networks. For example, both P17302 (Gap junction alpha-1 protein) and P43405 (Tyrosine-protein kinase SYK) rank high in the HD network but not in the HO network. As it is shown on Figure 2b, O14908 (PDZ domain-containing protein GIPC1) is the only protein among the three exclusive HO mediators that is part of the interaction network of HO mediators.

Additional File 2 shows the extracted GO terms of proteins ranked by different structural indices for the HD network, the HO network and the total network. For example, by considering the top 30 proteins ranked by degree in the HD network, we found that half of them are related to the processes 'intracellular signaling cascade' (GO:0007242) and 'protein amino acid phosphorylation' (GO:0006468), meanwhile in the HO network 16 of them are located in 'plasma membrane' (GO:0005886) and 13 of them are related to process 'cell surface receptor linked signal transduction' (GO:0007166).

The p-values of proteins quantify their average fit to the studied GO-terms (i.e. to what extent they can be characterized by certain functionality). By comparing those p-values to centrality and overlap indices used in this study, we can conclude that the performance of different indices vary strongly. In the total network, only the *TO*^3^_0.01 _index correlates significantly with biological function (Table 6). Note that the performance of *TO^3^_t _*depends on the *t *threshold used. Proteins in unique positions are, thus, typically involved in the above-mentioned key functions. The other relatively well-performing index is *CC*, whereas *D *and *BC *correlate with function only once each. *TI^3 ^*and *TO*^3^_0.005 _do not correlate with functions defined by GO terms. Furthermore, functional roles are best predictable by these structural indices in the HD network and less so for the HO network.

**Table 6:** Correlations between p-values and centrality.

	nD	nCC	nBC	TI^3^	TO^3^_0.01_	TO^3^_0.005_
HD/D	0.2568	**0.3077**	0.2635	0.2577	**0.3365**	0.2321
HD/TI	0.2467	0.2216	0.2062	0.1677	**0.4971**	0.3017
HD/TO	**0.3957**	**0.432**	**0.3993**	0.3775	0.3803	0.3386
	nD	nCC	nBC	TI^3^	TO^3^_0.01_	TO^3^_0.005_
HO/D	0.1501	0.144	0.1767	0.1925	0.1766	0.1487
HO/TI	0.0966	0.0007	0.0919	0.1127	0.1467	0.1254
HO/TO	0.3064	**0.4051**	0.3396	0.3211	0.3446	0.251
	nD	nCC	nBC	TI^3^	TO^3^_0.01_	TO^3^_0.005_
total/D	0.2687	0.2605	0.2136	0.1853	**0.4245**	0.3045
total/TI	0.2687	0.2605	0.2136	0.1853	**0.4245**	0.3045
total/TO	0.3734	0.3692	0.3599	0.3372	**0.4258**	0.3092

The Spearman rank correlation coefficients between the p-values of GO terms calculated for the most central nodes according to particular indices in particular networks and the node centrality values of the nodes. Bold numbers mean p < 0.05.

## Discussion

Based on its centrality ranks, P63104 (14-3-3 protein zeta/delta) corresponding to gene YWHAZ seems to be the second most important protein in these mediator processes. This is in concert with the literature, stating that P63104 is a chaperon [12] and is richly connected to several kinds of other molecules with mostly weak links [13]. Specifically, it is involved in cell growth and carcinogenesis [14], breast cancer reoccurrence after chemotherapy resistance [15], luteal sensitivity to PGF [16] and, finally, it is part of antiapoptotic (P13K/AKT) and cell proliferation (ERK/MAPK) pathways [17]. Its connecting position has been demonstrated by network analysis, showing its involvement in several HSNs (high-scoring subnetworks [18]). Ogihara et al. [19] suggested that the association with 14-3-3 protein may play a role in the regulation of insulin sensitivity by interrupting the association between the insulin receptor and IRS1. It means that P63104 probably mediate HD and HO through the regulation process of insulin (as insulin is a crucial hormone in human metabolic system). Typically it is not directly responsible for diseases (not assigned to any disease in the OMIM database) but very frequently mentioned as a candidate protein in the background, requiring further investigation [14].

The most important protein, P62993 (Growth factor receptor-bound protein 2) corresponding to gene GRB2 leads in all of the six structural importance ranks. It appears in the mammalian Grb2-Ras signaling pathway with SH2/SH3 domain interactions and several functions in embryogenesis and cancer [20]. Zhang et al. [21] also found that GRB2 is essential for cardiac hypertrophy and fibrosis in response to pressure overload and that different signaling pathways downstream of GRB2 regulate fibrosis, fetal gene induction, and cardiomyocyte growth. Yet, in the subgraph of the HO mediators, P62993 does not seem to occupy a central position but its phenotypic traits are likely to be affected through the links to non-mediators instead of other HO mediators. This kind of structural arrangement is advantageous for information integration, while a strongly connected mediator subnetwork implies functional redundancy.

Among the three exclusive HO (non-HD) mediators, O14908 (PDZ domain-containing protein GIPC1) corresponding to the gene GIPC1 appears in the HO mediator subgraph, while the other two are isolated (Q14232 - Translation initiation factor eIF-2B subunit alpha corresponding to gene EIF2B1; Q5JY77 - G-protein coupled receptor-associated sorting protein 1 corresponding to gene GPRASP1). This may suggest also that O14908 is an HD mediator. Its connection to heart disease is clear but its interaction with diabetes-related proteins is not documented in the OMIM databases (also not for the other two proteins). However, this inferred function is well supported by Klammt et al. [22] reporting on the role of O14908 in diabetes. A possible outcome of network analysis is to suggest potential updates in the databases.

The only protein that ranks higher than HD mediator proteins in the degree-based centrality rank of the HD network is P00533 (Epidermal growth factor receptor), corresponding to the gene EGFR. We could speculate that this protein might also mediate between H and D proteins. In the total PPI network, it is linked to two D proteins (Q9UQF2 - JNK-interacting protein 1; Q9UQQ2 - Signal transduction protein Lnk) but not to H protein. EGFR and its ligands are cell signaling molecules involved in a wide range of cellular functions, including cell proliferation, differentiation, motility, and tissue development [23]. Research on EGFR's pathogenesis have been focused on lung cancer [24] and have not discovered its link to heart diseases. However, Iwamoto and his colleagues observed the role of ErbB signaling in heart functions [25]. Also, it has been shown to be a central protein according to other sophisticated network analysis techniques [26], dominating the clique composition of certain pathways.

Based on our static, structural inference, it is not easy to decide whether a protein is „strongly linked to a disease" or it is a „disease protein". The definitions are very poor here. Is P00533 a H protein (causing heart diseases) or HD protein (mediating between H and D proteins)? The solution is to use inference for generating new hypotheses, improving databases and designing experiments, instead of regarding the inferred findings as results.

## Conclusions

Our study focused on only a few diseases but the approach and the methods used can be generalized. It may be interesting to extend this research to other diseases and to study the pleiotropic effects of mediators linking other disease pairs. The mediator proteins analyzed in this study typically have pleiotropic effects. They connect several pathways and influence several phenotypic traits. The reason why their inferred structural roles miss from the OMIM database is exactly that they act in a non-Mendelian way. They are typically not the singular elements of important pathways but weak connectors among several pathways of high importance. This way, their effects can be fundamental. Their understanding needs a multi-locus, systems-based, network view. As individual pathways are linked to networks, our non-Mendelian knowledge on linkage, epistasis and pleiotropy becomes larger. If network analysis makes these epistatic and pleiotropic effects quantifiable and predictable, we are getting closer to better understand delegated complexity [27]. From an application perspective, it would be interesting to see whether a healthy (intact and well-connected) network of mediators could contribute to healthy phenotypes or, in contrary, disconnecting the mediator network could be used to isolate diseases and reduce side-effects of drugs.

## Methods

### Data

We have analyzed human protein-protein interaction network (PPI) data extracted from the I2D database. I2D (Interologous Interaction Database) is an on-line database of known and predicted mammalian and eukaryotic protein-protein interactions [28]. It is one of the most comprehensive sources of known and predicted eukaryotic PPIs.

We carefully considered the completeness of the PPI network by investigating various human PPI databases. In their database, the Authors have collected data from almost all of the well-known human protein interaction databases including HRPD http://www.hprd.org/, BIND http://bind.ca/, MINT http://mint.bio.uniroma2.it/mint/ and Intact http://www.ebi.ac.uk/intact/, among others. Those databases are built by arrange of methods, some are experimental ones, some are predicted ones, and some are curated from the literature. By using the I2D database, we could thus construct the network integrated from multiple data sources. We investigated other databases not included in the I2D database, particularly the STRING database http://string.embl.de/ and we found that almost all high-scoring interactions in STRING were covered in our data set. Combining data from various sources is supposed to be more comprehensive for analyzing the PPI network than studying each data source separately. To obtain a more reliable set of protein interactions, we excluded all the interactions obtained by homology methods: only experimentally verified ones were included in our analysis. For the disease phenotypes, the clinical Online Mendelian Inheritance in Man database (OMIM, [29]) was investigated. We have checked whether we need to update our database used in Nguyen and Jordán [6] and found that we can use the same data set as the number of updates is negligible.

### Analysis

From the human PPI network data, we constructed: (1) a network of proteins mediating indirect effect between heart disease (H) and diabetes (D) proteins (i.e. HD mediators) and their direct neighbours (i.e. HD network); (2) a network of proteins mediating indirect effect between heart disease (H) and obesity (O) proteins (i.e. HO mediators) and their direct neighbours (i.e. HO network); and (3) an aggregated network of the two previous networks (i.e. total network). We considered only two-step mediator proteins, directly connected to two proteins related to different diseases and being otherwise unconnected (so, we do not consider chains of mediators). We have also studied the subnetworks of (1) and (2) without non-mediator neighbours. See Figure 1 for schematically illustrating the relationships between these five networks. Figure 2 shows the subnetworks without non-mediator neighbours (Figure 2a for HD and Figure 2b for HO). Figure 3a shows the HD and Figure 3b shows the HO network. The total network is shown in Figure 4.

Earlier we have determined the identity of these HD and HO mediators and quantified the strength of their mediator effect [6]. Here, we focus on the networks of mediators. Links in these networks are undirected (if protein *i *is linked to protein *j*, then *j *is also linked to *i*) and unweighted (we have no data for the intensity or strength of the interactions). We have characterized each network by some simple network statistics.

(i) The simplest index that provides the most local information about node *i *is its degree (*D_i_*). This is the number of other nodes connected directly to node *i*. We have calculated the normalized degree:

(1) $$nD_{i}=\frac{D_{i}}{N-1},$$

where *N *is the number of nodes in the network.

(ii) A measure of positional importance quantifies how frequently a node *i *is on the shortest path between every pair of nodes *j *and *k*. This index is called "betweenness centrality" (*BC_i_*) and it is used routinely in network analysis [30]. The normalized betweenness centrality index for a node *i *(*nBC_i_*) is:

(2) $$nBC_{i}\frac{2\times \underset{j<k}{\sum }g_{jk}\left(i\right)∕g_{jk}}{\left(N-1\right)\left(N-2\right)},$$

where *i *≠ *j *and *k*; *g_jk _*is the number of equally shortest paths between nodes *j *and *k*, and *g_jk _(i) *is the number of these shortest paths to which node *i *is incident (*g_jk _*may equal one). The denominator is twice the number of pairs of nodes without node *i*. This index thus measures how central a node is in the sense of being incident to many shortest paths in the network.

(iii) "Closeness centrality" (*CC_i_*) is a measure quantifying how short are the minimal paths from a given node *i *to all others [30]. The normalized index for a node *i *(*nCC_i_*) is:

(3) $$nCC_{i}=\frac{N-1}{\overset{N}{\underset{j=1}{\sum }}d_{ij}},$$

where *i*≠*j*, and *d_ij _*is the length of the shortest path between nodes *i *and *j *in the network. This index thus measures how close a node is to others. The larger *nCC_i _*is for node *i*, the more directly its deletion will affect the majority of other nodes.

(iv) Topological importance can also be quantified by general matrix algebra. In an undirected network, we define *a_n,ij _*as the effect of *j *on *i *when *i *can be reached from *j *in *n *steps. The simplest way of calculating *a_n,ij _*is when *n *= 1 (*i.e*. the effect of *j *on *i *in 1 step):

(4) $$a_{1,i,j}=\frac{1}{D_{i}},$$

where *D_i _*is the degree of node *i *(*i.e*. the number of its direct neighbours). We assume that indirect effects are multiplicative and additive. For instance, we wish to determine the effect of *j *on *i *in 2 steps, and there are two such 2-step pathways from *j *to *i*: one is through *k *and the other is through *h*. The effects of *j *on *i *through *k *is defined as the product of two direct effects (*i.e*. *a*_1,*kj*_×*a*_1,*ik*_), therefore the term multiplicative. Similarly, the effect of *j *on *i *through *h *equals to *a*_1,*hj*,1_×*a*_1,*ih*_. To determine the 2-step effect of *j *on *i *(*a*_2,*ij*_), we simply sum up those two individual 2-step effects:

(5) $$a_{2,ij}=a_{1,kj}\cdot a_{1,ik}+a_{1,hj}\cdot a_{1,ih},$$

and therefore the term additive. When the effect of step *n *is considered, we define the effect received by node *i *from all other nodes in the same network as:

(6) $$\psi_{n,i}=\overset{N}{\underset{j=1}{\sum }}a_{n,ij},$$

which is equal to 1 (*i.e*. each node is affected by the same unit effect.). Furthermore, we define the *n*-step effect originated from node *i *as:

(7) $$\sigma_{n,i}=\overset{N}{\underset{j=1}{\sum }}a_{n,ji},$$

which may vary among different nodes (*i.e*. effects originated from different nodes may be different). Here, we define the topological importance of node *i *when effects "up to" *n *step are considered as:

(8) $$TI_{i}^{n}=\frac{\overset{n}{\underset{m=1}{\sum }}\sigma_{m,i}}{n}=\frac{\overset{n}{\underset{m=1}{\sum }}\overset{N}{\underset{j=1}{\sum }}a_{m,ji}}{n},$$

which is simply the sum of effects originated from node *i *up to *n *steps (one plus two plus three...up to *n*) averaged over by the maximum number of steps considered (*i.e. n*). This *TI^n ^*index measures the positional importance of a node by considering how effects originated from such a given node can spread through the whole network to reach all nodes after a pre-defined *n *step length [31]. Calculations were performed by the CosBiLAB Graph software [32].

(v) Basically every node in a network is connected to each other, but it still matters how strongly they are connected (whether two nodes are neighbors in the network, second neighbors or more distant ones). Thus, it is of interest to study the indirect neighborhood of particular nodes, considering more than only the neighbors but less than the whole network. For a given step length *n *and a given network, there is an interaction matrix presenting the relative strengths of interactions between each pair of nodes *i *and *j*. We note that interaction strength is used here in a totally structural sense, with no dynamical component. If *n *exceeds 2 or 3, and the network is not very large, then there is non-zero interaction strength between each pair of nodes (everything is connected to everything else). Thus, an effect threshold (*t*) can be set, determining the "effective range" of the interaction structure of a given graph node *i*, and nodes within this effective range are defined as strong interactors of *i *(i.e. effects received from *i *being greater than *t*) whereas nodes outside this range are defined as *i*'s weak interactors (effects received from *i *is less than *t*). Since the sets of strong interactors of two or more nodes may overlap, it is possible to quantify this overlap (the number of shared strong interactors) in order to measure the positional uniqueness of individual graph nodes. The topological overlap between nodes *i *and *j *up to *n *steps (*TO^n^_t, ij_*) is the number of strong interactors appearing in both *i*'s and *j*'s effective ranges determined by the threshold *t*. The sum of all *TO*-values between node *i *and others provides the summed topological overlap of node *i*:

(9) $$TO_{t,i}^{n}=\overset{N}{\underset{j=1}{\sum }}TO_{t,ij}^{n}\left(i\neq j\right).$$

For simplicity of representation, we drop the subscript *i *for all indices. A more detailed description of this index can be found in [33]. Calculations were performed by the CosBiLAB Graph software [32]. Two thresholds have been used, t_1 _= 0.01 and t_2 _= 0.005.

Each of the six above mentioned structural indices were determined for every node in the networks. The 30 most central ones are presented for the HD network (Table 3), the HO network (Table 4) and the total network (Table 5). Additional File 2 presents all index values for all nodes in these networks.

Since different network indices provide different rankings, it is a question of how similar these rankings are. Similarity refers to robust importance ranks (irrespective to the index), while dissimilarity refers to the complementary information content of the different indices. For statistical analysis, we calculated the Spearman rank correlation coefficient for each pair of the indices in the three major networks (Table 1).

In order to better understand the ranking of nodal indices, we determined the distribution of each structural index for each network. We present these distributions for the total network in Figure 5. To test the significance of the observed rank correlation coefficients, we have constructed random networks. For each of our observed networks (i.e. HD, HO, total), we calculated the probability of two nodes being linked together:

(10) $$p=\frac{L}{\left(N^{2}-N\right)∕2}.$$

We have constructed 1000 random networks with fixed N and a *p *link probability. For each random network, we calculated the same centrality indices and determined the Spearman rank correlation coefficient for each pair of centrality indices. Since we have 1000 random networks, for each pair of centrality indices we thus have 1000 Spearman rank correlation coefficients. From their distribution, we determined the mean and the 95% confidence intervals. Results are summarized in Table 2.

For the top 30 nodes ranked by a particular index in a particular network, we quantified their biological function by calculating the p-values of GO terms [34]. Specifically, we determined the ratio of the top 30 nodes that can be characterized by a certain GO term and computed the associated p-values (Table 6). Bold numbers mean p < 0.05.

## Abbreviations

OMIM: Online Mendelian Inheritance in Man; PPI: protein-protein interaction; D: degree; BC: betweenness centrality; CC: closeness centrality; TI: topological importance; TO: topological overlap; GO: gene ontology

## Authors' contributions

TPN suggested the key idea, analyzed the database and wrote the paper. WCL contributed to analysis and wrote the paper. FJ made network analysis and wrote the paper. All authors read and approved the final manuscript.

## Supplementary Material

Additional file 1 **Network indices for three networks**. The values of the six network indices are given here for all nodes in the three networks. Click here for file

Additional file 2 **The GO terms and p-values studied in this paper**. The extracted GO terms and their statistics of proteins ranked by different structural indices for the HD network, the HO network and the total network. Click here for file
